# Conventional-Dose versus High-Dose Chemotherapy for Relapsed Germ Cell Tumors

**DOI:** 10.1155/2018/7272541

**Published:** 2018-03-15

**Authors:** Deaglan J. McHugh, Darren R. Feldman

**Affiliations:** ^1^Department of Medicine, Memorial Sloan Kettering Cancer Center, New York, NY, USA; ^2^Department of Medicine, Weill Medical College of Cornell University, New York, NY, USA

## Abstract

The majority of metastatic germ cell tumors (GCTs) are cured with cisplatin-based chemotherapy, but 20–30% of patients will relapse after first-line chemotherapy and require additional salvage strategies. The two major salvage approaches in this scenario are high-dose chemotherapy (HDCT) with autologous stem cell transplant (ASCT) or conventional-dose chemotherapy (CDCT). Both CDCT and HDCT have curative potential in the management of relapsed/refractory GCT. However, due to a lack of conclusive randomized trials, it remains unknown whether sequential HDCT or CDCT represents the optimal initial salvage approach, with practice varying between tertiary institutions. This represents the most pressing question remaining for defining GCT treatment standards and optimizing outcomes. The authors review prognostic factors in the initial salvage setting as well as the major studies assessing the efficacy of CDCT, HDCT, or both, describing the strengths and weaknesses that formed the rationale behind the ongoing international phase III “TIGER” trial.

## 1. Background

Germ cell tumors (GCTs), comprising 1% of male cancers and 5% of male genitourinary malignancies, are the most common tumor in young men. Most patients with advanced disease are cured with platinum-based chemotherapy; however, 20–30% patients will fail to achieve a durable response and require salvage treatment [1]. Unfortunately, the majority of patients requiring salvage chemotherapy will ultimately die, with death from GCT accounting for the greatest number of average life years lost of any non-childhood malignancy [2]. Presently, the two major salvage approaches include conventional-dose chemotherapy (CDCT) and high-dose chemotherapy (HDCT) with autologous stem cell transplant (ASCT).

Due to inconsistencies between retrospective and randomized data comparing these two strategies and the rarity of the patient population, a universally recommended approach in the initial salvage setting is lacking. As such, practices vary widely throughout the world and patients are highly encouraged to participate in clinical trials. The objective of this review is to outline the prognostic factors that affect outcome in the salvage setting, the data supporting both salvage chemotherapy strategies (CDCT and HDCT), and an ongoing randomized clinical trial that seeks to definitively establish one of these approaches as the standard of care in the initial salvage setting.

## 2. Prognostic Factors for Salvage Chemotherapy

For patients with metastatic GCT, prognostic factors at initial diagnosis are universally accepted with the International Germ Cell Cancer Cooperative Group (IGCCCG) classification used to guide first-line chemotherapy. Patients experiencing treatment failure with cisplatin-based first-line chemotherapy, however, represent a highly heterogeneous population. Traditionally, separate prognostic factor analyses were performed for patients undergoing CDCT and HDCT, respectively. Factors consistently associated with favorable outcome to salvage CDCT regimens across multiple series included gonadal primary tumor site, complete response (CR) to first-line chemotherapy, and disease-free interval after first-line chemotherapy of at least several months, whereas burden of disease and tumor marker levels at the time of salvage chemotherapy demonstrated prognostic importance in some but not all series [3–6].

Prognostic factors for outcome to salvage HDCT were initially reported on small number of patients at individual centers treated with one specific regimen limiting the generalizability of the findings. Beyer et al. were the first to develop a multicenter prognostic model for HDCT derived from 310 patients treated with various HDCT regimens at 4 different centers in Europe and the United States. Factors associated with an adverse outcome included primary mediastinal nonseminomatous germ cell tumor (PM-NSGCT), HCG ≥ 1,000 IU/L, progressive disease prior to HDCT, and platinum-refractory disease. A point value was assigned to each of these factors and used to calculate a cumulative score which separated patients into good, intermediate, and poor-risk groups with failure-free survival rates of 51%, 27%, and 5%, respectively.

In a retrospective study of 184 patients with gonadal or retroperitoneal primary GCTs (PM-NSGCT patients were excluded) treated with salvage high-dose carboplatin and etoposide at Indiana University (IU), Einhorn and colleagues identified platinum-refractory disease, IGCCCG poor-risk classification at first-line chemotherapy, and receipt of HDCT as third-line or later to be associated with adverse outcome [7]. Feldman et al. reported on 107 patients treated with salvage HDCT as part of a phase I/II study of the TI-CE regimen at Memorial Sloan Kettering Cancer Center (MSKCC) and observed PM-NSGCT, receipt of HDCT as third-line or later, presence of lung metastases, HCG ≥ 1,000 IU/L, ≥3 metastatic sites and IGCCCG intermediate- or poor-risk classification at first-line chemotherapy as associated with poor outcome [8]. A more recent study from IU of 364 patients treated with high-dose carboplatin and etoposide identified use of HDCT as third-line or later therapy (*n*=61, 17%), platinum-refractory disease (*n*=122, 34%), PM-NSGCT (*n*=20, 5%), nonseminoma histology (*n*=285, 78%), IGCCCG intermediate- or poor-risk disease (*n*=213, 59%) and HCG ≥ 1000 mIU/ml at HDCT initiation (*n*=90, 25%) as associated with adverse progression-free survival (PFS) [9].

Given the variation in prognostic factors reported in these and other studies, the separate focus on CDCT and HDCT, the limited number of patients from which these models were derived, and the small number of chemotherapy (particularly HDCT) regimens used, a collaborative effort, known as the International Prognostic Factor Study Group (IPFSG), was formed to develop a universally accepted prognostic model prior to initial salvage chemotherapy. The IPFSG collected data from a retrospective cohort of nearly 1,600 patients treated in 13 countries who progressed after initial cisplatin-based chemotherapy [10]. Fifty-one percent of these patients received HDCT, justifying the use of the classifier in this population. Prognostic variables on multivariate analysis applicable to the entire population independent of treatment approach (CDCT or HDCT) included primary tumor site, response to first-line treatment, progression-free interval between first-line therapy and relapse, tumor markers at relapse (AFP and HCG), and presence of liver, bone, or brain metastases at relapse. Each of these risk factors was assigned a numerical point value depending on its prognostic significance, with the sum total score (maximum of 10) used to segregate patients into five risk groups (very low, low, intermediate, high, and very high). Two-year PFS varied significantly according to risk group (very low risk: 91%, low risk: 64%, intermediate risk: 53%, high risk: 33%, and very high risk: 22%). Overall survival (OS) was also significantly different across the groups.

## 3. Salvage Conventional-Dose Chemotherapy

Cisplatin plus ifosfamide-containing regimens form the backbone of salvage CDCT, with studies testing a variety of third drugs to add to this combination. In the 1980s when the combination of cisplatin, vinblastine, and bleomycin (PVB) was the standard first-line regimen, the combination of etoposide, ifosfamide, and cisplatin (VIP) was the most common salvage regimen since it included two drugs not administered in the first-line setting. Once etoposide plus cisplatin (EP) and bleomycin plus EP (BEP) supplanted PVB as standard first-line treatment regimens, vinblastine, ifosfamide, and cisplatin (VeIP) became the most popular salvage regimen. Numerous studies reported on salvage VIP, VeIP, or both used in heterogeneous populations of patients including in the second-, third-, and even later-line setting. CR rates approximated 25–35% with durable remission rates of 5–15% [11–13]. Once activity was demonstrated, VeIP and VIP were moved forward for evaluation in the initial salvage setting with improvement in CR rates to approximately 40–50% and durable remission rates to approximately 25% (Table 1) [4, 5]. In the largest study evaluating VeIP as initial salvage treatment, Loehrer and colleagues treated 135 patients who experienced progressive GCT after first-line chemotherapy but who had remained disease-free for at least 3 weeks from their last chemotherapy dose and observed a CR rate of 50% and durable remission rate of 24% [5].

A subsequent phase I/II study at Memorial Sloan Kettering Cancer Center (MSKCC) evaluated the addition of paclitaxel to ifosfamide plus cisplatin (TIP) as initial salvage treatment of 46 patients [14]. This trial limited eligibility to patients with features predicting a favorable outcome to salvage CDCT including gonadal primary tumor site and either a CR to first-line chemotherapy or a partial response with negative tumor markers (PR-negative markers) lasting ≥6 months. The CR rate was 70%, 2-year PFS rate was 65%, and 63% of patients remained continuously disease-free with median follow-up of nearly 7 years. These significantly improved outcomes compared to initial salvage VeIP can be partially explained by favorable patient selection and a small number of patients included with metastatic disease in the setting of a second gonadal primary GCT. However, the study also included 14 (30%) patients with late relapse (>2 years from the end of prior chemotherapy), a group with historically poor outcome to salvage CDCT, with 7 achieving durable remissions. TIP has also been studied as salvage therapy in series with less restrictive eligibility criteria and using a lower dose of paclitaxel and in some cases, also ifosfamide [15, 16]. Not surprisingly, these studies did not duplicate the high CR and durable remission rates achieved in the MSKCC TIP series, although Mardiak observed a 65% objective response rate (ORR), 41% CR rate, and 47% 2-year PFS rate, all of which still compare favorably to VeIP [15].

More recently, Fizazi et al. reported on a phase II trial combining gemcitabine with ifosfamide and cisplatin (GIP) in the initial salvage treatment of 37 patients with GCT and the favorable criteria used in the MSKCC TIP study [17]. Although this trial did not reach its primary endpoint of a 65% CR rate, GIP still demonstrated activity with favorable response (e.g., CR or PR-negative markers), CR, and 2-year PFS rates of 78%, 54%, and 51%, respectively. The authors suggested GIP exhibited similar efficacy as TIP in this favorable-risk population but with less febrile neutropenia (22% versus 48%) and severe neurotoxicity (0% versus 7%). However, the durable remission rate was lower with GIP even though none of the patients had experienced a late relapse.

Given the traditional ifosfamide-cisplatin backbone of curative salvage CDCT regimens, use of VIP as an alternative to BEP for first-line treatment of intermediate- and poor-risk patients can make selecting an initial salvage CDCT regimen difficult. This is particularly applicable to patients > 50 years old or those with pulmonary compromise at diagnosis, groups with increased susceptibility to bleomycin lung toxicity in whom bleomycin is often avoided. Potential options include PVB (if patients are now candidates for bleomycin) given neither vinblastine nor bleomycin was used in the first-line setting, or the combination of gemcitabine, oxaliplatin, and paclitaxel (GOP), which can also result in some durable CRs [18]. However, a more common approach seems to be initial salvage HDCT with ASCT.

A lack of phase III studies demonstrating the superiority of any one conventional-dose regimen has led to variation in practice. Nevertheless, TIP appears to be the most commonly used regimen, perhaps because no study has yet to report a higher durable remission rate than the MSKCC TIP series and given the study of GIP did not meet its primary endpoint [17].

## 4. Salvage High-Dose Chemotherapy

The concept of HDCT stemmed from work in the 1980s, demonstrating that tumor cell resistance acquired after therapy with alkylating agents could be overcome by dose intensification (e.g., increase in dose by multiples of 5–10). To avoid the need for direct bone marrow harvest, growth factor support, typically G-CSF with or without chemotherapy, is used to disrupt adhesions between hematopoietic stem cells (HSCs) and stromal cells in the bone marrow, releasing HSCs into the vasculature for collection.

An early report by Nichols et al. demonstrated the potential for HDCT to salvage some patients with relapsed GCT but at the cost of significant toxicity, including treatment-related mortality (TRM) [19]. With improved supportive care measures, four modern studies provide robust evidence of clinical benefit with salvage HDCT with limited TRM (Table 2) [7–9, 20]. In a retrospective study at IU [7], 184 consecutive patients with testicular or retroperitoneal primary GCT underwent two cycles of high-dose carboplatin and etoposide, each followed by ASCT. Carboplatin dose was calculated using body surface area (BSA), and patients in remission after HDCT received adjuvant oral etoposide for 3 months. At median follow-up of 48 months, 116 (63%) patients had a durable CR with 5-year OS of 65%. Notably, patients with PM-NSGCT and late relapse were excluded from this series.

MSKCC investigators reported outcomes with the TI-CE regimen within a prospective phase I/II trial of 107 patients with relapsed/refractory GCT and unfavorable features for achieving a durable remission to CDCT including an extragonadal primary site, incomplete response to initial therapy or relapse/incomplete response to salvage CDCT [8]. Patients received two cycles of paclitaxel with ifosfamide every two weeks, followed by 3 cycles of high-dose carboplatin and etoposide every three weeks. In contrast to the IU study, carboplatin dose was based on area under the curve (AUC) instead of BSA and patients with late relapses and PM-NSGCT were eligible. Fifty percent (*n*=54) of patients achieved a CR and 8% (*n*=8) achieved a PR-negative marker response. At median follow-up of 61 months, 5-year disease-free survival (DFS) and 5-year OS were 47% and 52%, respectively. When only patients who would have met the criteria for HDCT at Indiana University were considered, the 5-year DFS and OS improved to 57% and 62%, respectively [8]. In addition, the data demonstrated that patients with late relapse and PM-NSGCT remain potentially curable with salvage HDCT, albeit with a lower probability.

The IU group recently reported on another 364 patients treated with salvage HDCT and ASCT between 2004 and 2014 [9]. A notable difference from their prior series was inclusion of patients with PM-NSGCT; patients with late relapse were still excluded. Treatment was identical to the prior IU series with two consecutive courses of 700 mg/m^2^ carboplatin and 750 mg/m^2^ etoposide daily for 3 consecutive days, followed by ASCT. Maintenance oral etoposide was administered to 134 patients who achieved a CR, of whom 105 received three cycles. With median follow-up of 3.3 years, the 2-year PFS and OS were 60% and 66%, respectively. Of 9 treatment-related deaths (2.5%), 6 occurred within 30 days of HDCT completion and three resulted from secondary leukemia. The authors acknowledged the potential for selection bias that is inherent within studies of HDCT-eligible populations.

The German Testicular Cancer Study Group compared sequential HDCT with single HDCT in 211 patients with relapsed or refractory GCT. Patients were randomized to one cycle of VIP plus three cycles of high-dose carboplatin and etoposide (arm A) or three cycles of VIP plus one cycle of high-dose carboplatin, etoposide, and cyclophosphamide (arm B), followed by ASCT. At a median of 7.5 years of follow-up, 5-year PFS and OS rates were 48% versus 46% and 50% versus 40%, respectively, for sequential and single HDCT, respectively. The 10% difference in 5-year OS, which almost reached statistical significance (*p*=0.057) was attributed to the fewer treatment-related deaths in the sequential HDCT arm (4% versus 16%). When IPFSG scores were applied, the improved outcome with sequential HDCT was most prominent in IPFSG high-risk patients treated in the first salvage setting (3-year OS 56% versus 11%) and those attempting HDCT as second salvage (3-year OS 40% versus 20%) [20].

## 5. HDCT versus CDCT as First Salvage Therapy

Given the efficacy of both CDCT and HDCT in the salvage setting and in particular, the excellent outcomes with HDCT for patients with unfavorable features, several studies have attempted to compare these two strategies to establish the optimal initial salvage approach (Table 3). In the first study to address this question, Beyer et al. conducted a retrospective, matched pair analysis comparing initial salvage HDCT and CDCT [21]. Fifty-five pairs of patients with relapsed/refractory NSGCT treated with either initial salvage CDCT or HDCT between 1981 and 1995 had full matches on at least 4 of 5 selected prognostic factors (primary tumor location, response to first-line treatment, duration of response, and serum AFP and serum HCG). Hazard ratios favored HDCT for both event-free survival (EFS; 0.72–0.84) and OS (0.77–0.83). Results remained consistent when restricting analysis to those who received both etoposide and cisplatin as initial therapy. Limitations were acknowledged, including the fact that CDCT patients were treated at multiple institutions as part of a cooperative group trial and from earlier time points, in contrast to HDCT, where patients were treated at a single center and more recently. These differences were previously demonstrated to be of prognostic significance [22, 23]. Furthermore, 18% of CDCT patients did not receive etoposide in their first-line regimen and not all patients received ifosfamide during salvage CDCT. Finally, selection bias leading to patients with better performance status or fewer comorbidities receiving initial salvage HDCT could not be excluded. Collectively, these weaknesses may overinflate the differences favoring HDCT.

In the only randomized phase III study to address this question, a multi-institutional European trial (IT-94) compared four cycles of VIP or VeIP (arm A) with three such cycles followed by one cycle of high-dose carboplatin, etoposide, and cyclophosphamide with ASCT (arm B) [24]. Of 263 eligible patients, 128 and 135 patients were randomized to arms A and B, respectively, with 103 (80%) and 98 (73%) receiving all four chemotherapy cycles, respectively. Among evaluable patients, objective response (OR) rates after 4 cycles were 67% versus 75% (*p*=0.23). At median follow-up of 45 months, there was no significant difference in EFS or OS, though there was a significant difference in 3-year EFS (55% versus 75%) favoring HDCT among patients achieving a CR. Although the authors concluded that IT-94 demonstrated no clinical benefit with initial salvage HDCT, there are several notable limitations of this study. Accrual was lower than expected with early stoppage of the study negatively impacting the statistical power. Only a single (rather than sequential) HDCT cycle was administered as part of arm B and 27% of patients randomized to HDCT did not receive the fourth (high-dose) cycle. Mortality was also higher than expected at 7% for Arm B (versus 3% for Arm A), potentially obscuring any benefit with HDCT. Furthermore, patients with incomplete response to initial therapy were excluded, a group more likely to benefit from HDCT given historically poor outcomes with salvage CDCT.

In light of these limitations and the small size of the prior Beyer-matched pairs analysis, Lorch et al. used the IPFSG database to retrospectively compare initial salvage HDCT and CDCT among 1594 patients with progression after at least three cisplatin-based cycles in the first-line setting. Two-year PFS was significantly superior after HDCT (*n*=821) compared with CDCT (*n*=773), both overall (50% versus 28%, *p* < 0.001) and within each IPFSG risk category. There was also a significant improvement in 5-year OS (53% versus 41%, *p* < 0.001) favoring initial salvage HDCT, overall, and within each IPFSG subgroup with the exception of low-risk patients. Despite the benefit observed for HDCT, numerous biases were acknowledged including patient selection bias (given the nonrandom treatment allocation) potentially favoring HDCT, wide variation in the CDCT regimens used, with some possibly inferior to others (e.g., TIP), and potential investigator bias in considering patients to progress earlier with CDCT than HDCT. Given the inherent methodological limitations of this retrospective, albeit large analysis, this study does not definitively prove the superiority of HDCT over CDCT, but rather underscores the need for a prospective randomized trial to compare a highly effective CDCT regimen (TIP) to a sequential HDCT regimen (TI-CE), and this ultimately formed the foundation of the TIGER trial.

## 6. The TIGER Trial

**Figure 1:**
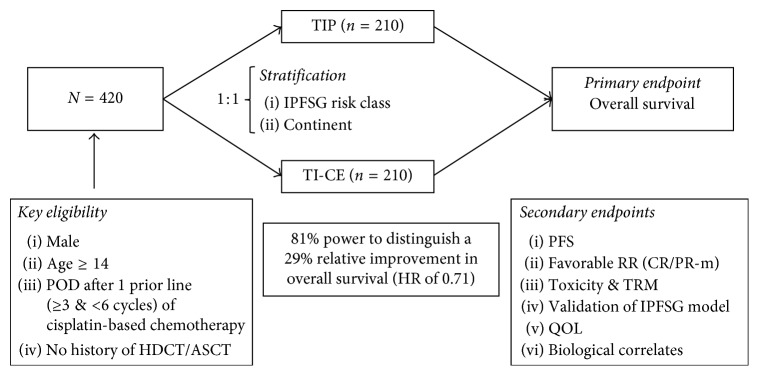
Study design of the TIGER trial (Alliance 0311012; EORTC 1407). POD, progression of disease; HDCT/ASCT, high-dose chemotherapy with autologous stem cell transplant; TIP, paclitaxel, ifosfamide, and cisplatin; TI-CE, paclitaxel and ifosfamide followed by high-dose carboplatin and etoposide; IPFSG, International Prognostic Factors Study Group; PFS, progression-free survival; response rate; CR, complete response; PR-m, partial response with normal tumor markers; TRM, treatment-related mortality; QOL, quality of life.

The TIGER trial (A031102, E1407) is an international collaboration among many centers in North America, Europe, and Australia with the goal of determining the optimal initial salvage chemotherapy approach in patients with advanced GCT. Patients with unequivocal disease progression after a minimum of 3 and no more than 6 cisplatin-based chemotherapy cycles, administered in the first-line setting, are randomized 1:1 to receive CDCT with TIP (control arm) or HDCT using TI-CE (experimental arm) as illustrated in Figure 1. The primary endpoint is OS, with secondary endpoints including PFS, favorable response rate, toxicity, quality of life, and biological correlates. Patients will be stratified by a modification of their IPFSG category into low-, intermediate-, and high-risk groups, with prospective evaluation of outcomes by risk group. With a target accrual of 420 patients, the study is powered to detect a 29% difference in OS between the two arms. The study is ongoing and as of 11/1/2017 has accrued 67 (16%) patients. Results are anxiously awaited and will hopefully definitively establish either HDCT or CDCT as the standard of care in the initial salvage setting.

## 7. Conclusion

Both CDCT and HDCT have curative potential in the salvage management of relapsed/refractory GCT. Common salvage CDCT regimens include VeIP, TIP, and GIP, with no randomized data establishing one clearly superior regimen, although the best results reported to date are with TIP, albeit in a favorably selected patient population. Salvage HDCT regimens can achieve durable remissions even in patients with unfavorable characteristics with low TRM. As a result of conflicting data from retrospective series suggesting improved outcomes with HDCT and the IT-94 randomized study demonstrating no benefit to HDCT over CDCT, the optimal initial salvage approach remains unclear with practices varying widely around the world. The global cooperative group-led TIGER trial (A031102, E1407) is testing TIP versus TI-CE in this setting and seeks to definitively answer this important question.

## Figures and Tables

**Table 1:** Prospective studies examining the use of initial salvage conventional-dose chemotherapy.

Author (year)	N	CDCT Regimen(s)	Notable inclusion or exclusion criteria	EP/BEP as first-line therapy	CR/PR to first-line therapy	IR to first-line therapy	CR	Median f/u (months)	Durable remission
McCaffrey et al. [4]	56	VeIP or VIP	None	53%	36%	64%	36%	52	23%
Loehrerr et al. [5]	135	VeIP	Cisplatin-refractory patients excluded^a^	100%	100%	0%	50%	72^b^	24%
Kondagunta et al. [14]	46	TIP	Included only patients with CR- or PR-negative marker to first line, gonadal primary, and <6 cycles of cisplatin in first line	74%	100%	0%	70%	69	63%
Fizazi et al. [17]	37	GIP	Included only patients with CR or PR-negative marker to first line, gonadal primary, and <6 cycles of cisplatin in first line	86%	100%	0%	54%	53	51%

CDCT, conventional-dose chemotherapy; EP, etoposide plus cisplatin; BEP; bleomycin, etoposide, and cisplatin; IR; incomplete response; CR, complete response; f/u, follow-up; VIP, etoposide, ifosfamide, and cisplatin; VeIP, vinblastine, ifosfamide, and cisplatin; GIP, gemcitabine, ifosfamide, and cisplatin; ^a^progression at <3 weeks after completion of first-line chemotherapy; ^b^minimal (not median) follow-up.

**Table 2:** Studies examining the use of high-dose chemotherapy as initial (or later) salvage therapy.

Author (Year)	Study design	N	Notable I/E criteria	Median f/u (m)	HDCT as initial salvage	HDCT regimen	Cycles	Durable CR	OS
Einhorn et al. [7]	Retrospective	184	I: None E: PM-NSGCT and late relapse	48	73%	Carboplatin 700 mg/m^2^ (d 1–3) Etoposide 750 mg/m^2^ (d 1–3)	2	58% 2-year DFS	65% at 5 years
Feldman et al. [8]	Prospective, phase I/II	107	I: ≥1 adverse prognostic feature for salvage CDCT^a^ E: None	61	76%	Part A (TI): Paclitaxel 200 mg/m^2^ (d 1) Ifosfamide 2000 mg/m^2^ (d 1–3)	2	48% 5-year DFS	52% at 5 years
						Part B (CE): Carboplatin AUC 7-8 (d 1–3) Etoposide 400 mg/m^2^ (d 1–3)	3		
Lorch et al. [20]	Prospective, randomized phase III	211	I: None E: None	90	86%	Arm A: VIP	1	52% 2-year PFS	50% at 5 years
						Carboplatin 500 mg/m^2^ (d 1–3) Etoposide 500 mg/m^2^ (d 1–3)	3		
						Arm B: VIP	3	47% 2-year PFS	40% at 5 years
						Carboplatin 550 mg/m^2^ (d 1–4) Etoposide 600 mg/m^2^ (d 1–4) Cyclophosphamide 1600 mg/m^2^ (d 1–4)	1		
Adra et al. [9]	Retrospective	364	I: None E: Late relapse	40	83%	Carboplatin 700 mg/m^2^ (d 1–3) Etoposide 750 mg/m^2^ (d 1–3)	2	60% 2-year PFS	66% at 2 years

I, inclusion; E, exclusion; HDCT, high-dose chemotherapy; f/u, follow-up; m, months; CR, complete response; OS, overall survival; PM-NSGCT, primary mediastinal nonseminomatous germ cell tumor; d, day; DFS, disease-free survival; AUC, area under the curve; PFS, progression-free survival; VIP, etoposide, ifosfamide, and cisplatin; ^a^extragonadal primary site, incomplete response (IR) to first-line therapy, and PD after a salvage CDCT (cisplatin plus ifosfamide-based) regimen.

**Table 3:** Studies comparing the use of conventional-dose chemotherapy with high-dose chemotherapy as initial salvage therapy.

Author (Year)	Study design	Notable I/E criteria	Treatment regimen	NCDCT versus HDCT	Median f/u	PFS/EFS	OS
Beyer et al. [21]	Retrospective matched-pair analysis^a^	I: NSGCT only E: Pure seminoma	CDCT: Any HDCT: VIP × 2–3 then 1 cycle ICE	55^b^ 55^b^	7.5y & 9y^c^ 5y	HR 0.72–0.84^d^	HR 0.77–0.83^d^
Pico et al. [24]	Phase III randomized (IT-94)	I: none E: IR to first-line therapy; pure seminoma treated with carboplatin	CDCT: VIP or VeIP × 4 HDCT: VIP or VeIP × 3 then CarboPEC × 1	128 135	45 months	35% at 3y 42% at 3y	47% 47%
Lorch et al. [20]	Retrospective, including IPFSG subgroup analyses	I: ≥3 cycles of EP-based CT E: cisplatin-refractory disease^e^	CDCT: Any HDCT: ≥1 cycle carboplatin + etoposide ± ifosfamide, thiotepa, or cyclophosphamide	773 821	58 months	HR 0.44^d^	HR 0.65^d^

I, inclusion; E, exclusion; CDCT, conventional-dose chemotherapy; HDCT, high-dose chemotherapy; f/u, follow-up; PFS, progression-free survival; EFS, event-free survival; OS, overall survival; NSGCT, nonseminomatous germ cell tumor; VIP, etoposide, ifosfamide, and cisplatin; ICE, ifosfamide, carboplatin, and etoposide; y, years; HR, hazard ratio; CarboPEC, carboplatin, etoposide, and cyclophosphamide; IPFSG, International Prognostic Factor Study Group; ^a^matching factors: primary tumor location, response to first-line treatment, duration of response, HCG and AFP levels; ^b^fifty-five pairs of patients had full matches on >4 of 5 factors; ^c^median follow-up for patients treated at Medical Research Council and Munich, respectively; ^d^hazard ratio(s) favoring HDCT; ^e^progression within 4 weeks of the first-line cisplatin-based regimen.
